# Effects of multimodal analgesia with combined quadratus lumborum block on enhanced recovery after Kasai portoenterostomy in infants with biliary atresia: a retrospective cohort study

**DOI:** 10.3389/fped.2026.1843140

**Published:** 2026-05-21

**Authors:** Jing Ji, Haijing Zhang, Zishi Liu, Siyi Yan, Zhifeng Gao

**Affiliations:** Department of Anesthesiology, Beijing Tsinghua Changgung Hospital, School of Clinical Medicine, Tsinghua Medicine, Tsinghua University, Beijing, China

**Keywords:** biliary atresia, infant, Kasai surgery, multimodal analgesia, quadratus lumborum block, retrospective cohort study

## Abstract

**Background:**

Biliary atresia is a severe hepatobiliary disease in infancy, and Kasai surgery is the standard surgical treatment. However, the marked postoperative pain and opioid-related adverse effects may affect early recovery. This study aimed to evaluate the effect of multimodal analgesia combined with quadratus lumborum block (QLB) on early recovery after Kasai surgery in infants with biliary atresia.

**Methods:**

In this single-center retrospective cohort study76 infants who underwent Kasai surgery were included, of whom 39 received general anesthesia combined with ultrasound-guided bilateral QLB (GA + QLB group) and 37 received general anesthesia alone (GA group). The primary outcome was postoperative extubation time. Secondary outcomes included intraoperative opioid exposure expressed as morphine milligram equivalents (MME), FLACC pain scores at 2, 6, 12, and 24 h after surgery, postoperative analgesic strategy in the ICU, postoperative nausea and vomiting (PONV), and other recovery indicators. Linear mixed-effects models were used to analyze the postoperative pain trajectory, and inverse probability of treatment weighting (IPTW) and multivariable regression were used for sensitivity analyses.

**Results:**

Compared with the GA group, the GA + QLB group had a significantly shorter postoperative extubation time [5.00 (3.00, 10.50) vs 24.00 (20.00, 35.00) min, *P* < 0.001], lower intraoperative MME [10.80 (7.44, 15.06) vs 18.00 (13.50, 18.00) mg, *P* < 0.001], lower FLACC scores at rest and during activity within 24 h after surgery (all *P* < 0.001), and a lower incidence of PONV [23.1% vs 54.1%, *P* = 0.009]. After adjustment for postoperative analgesic strategy in the ICU, QLB remained independently associated with faster extubation time, lower intraoperative MME, and a lower risk of PONV.

**Conclusion:**

In infants undergoing Kasai surgery for biliary atresia, multimodal analgesia combined with quadratus lumborum block was associated with faster extubation, reduced intraoperative opioid exposure, improved postoperative pain control, and a lower incidence of PONV. This strategy may provide an effective and safe perioperative analgesic option for this vulnerable population.

## Introduction

Biliary atresia is a severe cholestatic disease in infancy and one of the most important causes of pediatric liver transplantation worldwide (1). Kasai surgery is the standard treatment for biliary atresia, and its success largely determines the short-term prognosis and the possibility of preserving the native liver (2). However, the procedure is highly traumatic, and the postoperative pain burden remains substantial. Opioids are still the mainstay of analgesia after abdominal surgery, but in infants they may lead to respiratory depression, delayed extubation, postoperative nausea and vomiting, and delayed recovery of gastrointestinal function (3). Therefore, it is especially important to explore perioperative analgesic strategies that can effectively relieve pain while reducing opioid exposure.

Quadratus lumborum block (QLB) is an ultrasound-guided fascial plane block that has received increasing attention in abdominal surgery in recent years (4, 5). In pediatric abdominal surgery, QLB has shown promising analgesic efficacy, reducing postoperative pain scores and opioid requirements and improving recovery quality (6–8). Compared with neuraxial techniques, QLB is easier to perform in some small infants, avoids puncture of the epidural space, and theoretically has a lower risk of hematoma and infection. However, evidence regarding the application of QLB in Kasai surgery for biliary atresia, especially in infants, remains very limited.

At present, evidence for regional analgesia in Kasai surgery mainly comes from epidural analgesia and small-sample case reports (9–11). Whether QLB can provide benefits similar to or better than those of epidural analgesia in this operation, while maintaining a favorable safety profile, still lacks direct evidence. Therefore, this study retrospectively analyzed infants who underwent Kasai surgery at our center, comparing general anesthesia combined with bilateral ultrasound-guided QLB with general anesthesia alone, in order to evaluate its association with postoperative extubation time, pain control, opioid exposure, and complications, and to provide a clinical basis for perioperative analgesic optimization in infants with biliary atresia.

## Materials and methods

### Study design and setting

**Figure 1:**
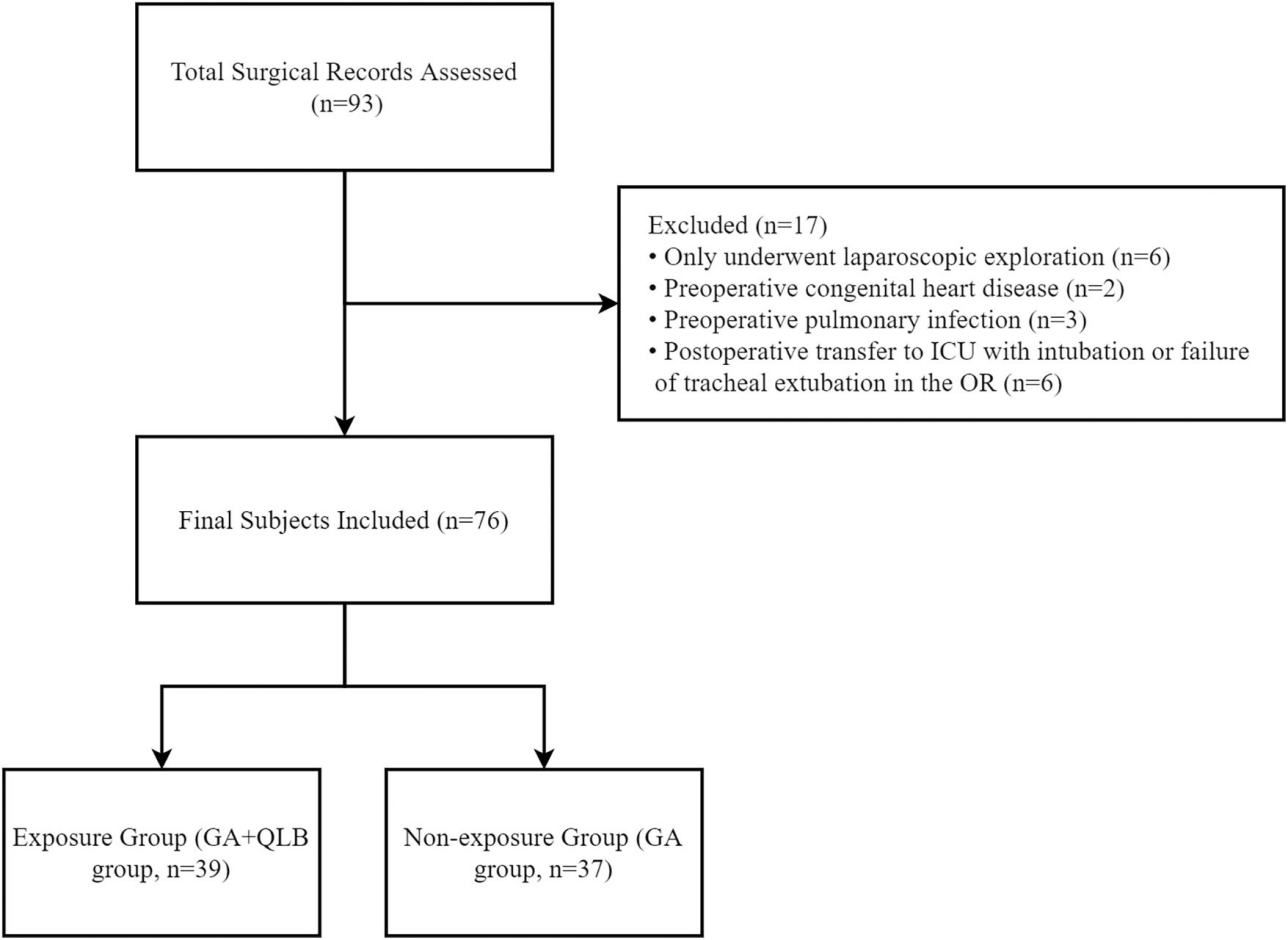
Flowchart of participant inclusion.

This was a single-center retrospective cohort study. Infants who underwent Kasai surgery in Beijing Tsinghua Changgung Hospital from July 2023 to March 2025 were screened. The inclusion criteria were: (1) age < 1 year; (2) diagnosis of biliary atresia and completion of Kasai surgery under general anesthesia; and (3) complete perioperative anesthesia and postoperative follow-up records. The exclusion criteria were: (1) combined with severe congenital heart disease or other major organ malformations; (2) preoperative liver failure or severe coagulation dysfunction; (3) emergency surgery or concomitant major surgery during the same admission; and (4) missing key variables or incomplete records. According to whether bilateral ultrasound-guided QLB was performed intraoperatively, the patients were divided into the GA + QLB group and the GA group. The participant inclusion flow is shown in Figure 1.

### Anesthesia management and QLB procedure

All infants underwent standard monitoring after entering the operating room and received general anesthesia induction and endotracheal intubation. Intraoperative anesthetic management followed the routine practice of the attending anesthesiologist. In the GA + QLB group, after completion of general anesthesia induction and intubation, bilateral anterior QLB was performed under ultrasound guidance.

The procedure was as follows: after induction of general anesthesia and endotracheal intubation, the infant was placed in the supine position. A linear ultrasound probe was placed parallel to the posterior axillary line at the level of the umbilicus and then moved slightly caudally from the lower pole of the kidney to identify the abdominal wall muscle layers, quadratus lumborum, and psoas major. An in-plane approach was used, and the needle was advanced from anteromedial to posterolateral to the anterior fascial plane of the quadratus lumborum (between the quadratus lumborum and psoas major). After injection of 0.1% ropivacaine at 1 mL/kg per side, separation of the fascial plane and spread of the solution along the target compartment were observed. Because this was a retrospective study, unified awake sensory plane testing or onset-time records could not be obtained in all infants. Therefore, “clear identification of anatomical landmarks under ultrasound, spread of the injectate along the target fascial plane, and no intraoperative record of block failure or conversion to other regional analgesic measures” were used as the operational criteria for technical success.

### Outcomes and variable definitions

The primary outcome was postoperative extubation time, defined as the time from the end of surgery to extubation. Secondary outcomes included: (1) FLACC pain scores at 2, 6, 12, and 24 h after surgery under resting and active conditions; (2) intraoperative opioid exposure; (3) ICU postoperative analgesic strategy within 24 h; (4) postoperative nausea and vomiting, pruritus, postoperative complications, duration of urinary catheterization, time to enteral feeding recovery, time to jaundice regression, length of hospital stay, and perioperative hypothermia.

Intraoperative opioid exposure was uniformly expressed as morphine milligram equivalents (MME, mg; calculated as oral morphine equivalents). For cases in which equivalent conversion had already been completed in the electronic anesthesia record, the MME value was extracted directly; if the original record contained the drug name and dose, the values were standardized according to the departmental preset conversion rules before inclusion in the analysis. The preset conversion rules used in this study were as follows: 10 mg oral morphine was defined as 10 MME; 30 mg oral morphine ≈ 10 mg intravenous morphine; 100 mg intramuscular tramadol = 30 MME; 5 mg intramuscular dezocine = 15 MME; 10 μg sufentanil = 30 MME; and 100 μg intravenous fentanyl = 30 MME. These conversions were used only for the statistical comparisons in this study and not as a basis for clinical dose interchange.

Postoperative analgesic strategies were classified as active analgesia and non-active analgesia. Active analgesia referred to postoperative use of opioids or continuous analgesic pump treatment in the ICU within 24 h, whereas non-active analgesia referred to no routine analgesic intervention or only symptomatic treatment as needed.

### Statistical analysis

Continuous variables with an approximately normal distribution are presented as mean ± standard deviation and were compared using the Student's t-test. Non-normally distributed continuous variables are presented as median [interquartile range (Q1, Q3)] and were compared using the Mann–Whitney U test. Categorical variables are presented as counts (percentages) and were compared using Pearson's chi-square test or Fisher's exact test, as appropriate. Because postoperative opioid administration in the ICU could influence outcomes, its effect was incorporated into multivariable linear regression models for postoperative extubation time, intraoperative MME, and FLACC scores at rest and during activity.

## Results

### Participants and baseline characteristics

A total of 76 eligible infants were included, including 39 in the GA + QLB group and 37 in the GA group; the numbers of neonates aged ≤ 30 days were 18 and 21, respectively. There were no statistically significant differences between the groups in baseline age, sex, body weight, body length, or preoperative liver function indices. Intraoperative blood loss, urine output, and operative duration were also comparable (Table 1).

**Table 1:** Comparison of baseline characteristics and perioperative variables between the two groups.

Variable	GA + QLB group (*n* = 39)	GA group (*n* = 37)	*P* value
Age (days)	34.00 (21.00, 52.00)	26.00 (18.00, 50.00)	0.360
Female, n (%)	23 (59.0)	22 (59.5)	1.000
Weight (kg)	3.90 (3.50, 4.70)	4.00 (3.50, 4.50)	0.823
Length (cm)	53.00 (51.50, 56.50)	53.00 (51.00, 55.00)	0.501
Preoperative ALT (U/L)	69.40 (26.00, 124.75)	41.00 (22.00, 92.00)	0.391
Preoperative AST (U/L)	112.00 (54.00, 193.55)	81.00 (49.00, 192.00)	0.436
Preoperative total bilirubin (μmol/L)	158.80 (128.95, 199.75)	146.00 (131.00, 194.00)	0.629
Preoperative direct bilirubin (μmol/L)	102.80 ± 36.16	100.43 ± 35.17	0.773
Intraoperative blood loss (mL)	5.00 (5.00, 10.00)	5.00 (5.00, 5.00)	0.628
Intraoperative urine output (mL)	35.00 (20.00, 70.00)	30.00 (20.00, 50.00)	0.572
Operative duration (min)	154.00 ± 37.72	148.35 ± 23.71	0.435

Approximately normally distributed variables are presented as mean ± standard deviation, skewed variables as median (Q1, Q3), and categorical variables as number (percentage). ALT, alanine aminotransferase; AST, aspartate aminotransferase.

### Primary outcome: postoperative extubation time

Postoperative extubation time was significantly shorter in the GA + QLB group than in the GA group [5.00 (3.00, 10.50) min vs. 24.00 (20.00, 35.00) min, *P* < 0.001]. After IPTW, all absolute SMDs were < 0.10, indicating good covariate balance after weighting (maximum 0.051). In the IPTW analysis, the GA + QLB group still had a shorter postoperative extubation time (adjusted mean difference, −21.98 min; 95% CI, −28.87–−15.10). Multivariable regression yielded consistent results, with an adjusted mean difference of −21.93 min (95% CI, −28.68–−15.18) (Table 2).

**Table 2:** Postoperative extubation time.

Panel A. Unadjusted comparison			
Variable	GA + QLB group (*n* = 39)	GA group (*n* = 37)	*P* value
Postoperative extubation time (min)	5.00 (3.00, 10.50)	24.00 (20.00, 35.00)	< 0.001
Panel B. Adjusted analyses			
Model	Effect estimate	95% CI/balance	*P* value
Propensity score balance	All absolute SMDs after weighting < 0.10 (maximum 0.051)		
IPTW model	Adjusted mean difference −21.98 min	95% CI −28.87–−15.10	< 0.001
Multivariable model	Adjusted mean difference −21.93 min	95% CI −28.68–−15.18	< 0.001

IPTW, inverse probability of treatment weighting; CI, confidence interval; SMD, standardized mean difference.

### Intraoperative opioid exposure

Intraoperative MME was significantly lower in the GA + QLB group than in the GA group [10.80 (7.44, 15.06) mg vs. 18.00 (13.50, 18.00) mg, *P* < 0.001]. After IPTW, intraoperative MME remained lower in the GA + QLB group (adjusted mean difference, −5.67 mg; 95% CI, −9.21–−2.10). The multivariable model showed a similar result (adjusted mean difference, −5.55 mg; 95% CI, −9.30–−1.80) (Table 3).

**Table 3:** Intraoperative opioid exposure.

Panel A. Unadjusted comparison			
Variable	GA + QLB group (*n* = 39)	GA group (*n* = 37)	*P* value
Intraoperative opioid exposure (MME, mg)	10.80 (7.44, 15.06)	18.00 (13.50, 18.00)	< 0.001
Panel B. Adjusted analyses			
Model	Effect estimate	95% CI/balance	*P* value
Propensity score balance	All absolute SMDs after weighting < 0.10 (maximum 0.051)		
IPTW model	Adjusted mean difference −5.67 mg	95% CI −9.21–−2.10	0.002
Multivariable model	Adjusted mean difference −5.55 mg	95% CI −9.30–−1.80	0.004

MME, morphine milligram equivalents; IPTW, inverse probability of treatment weighting; CI, confidence interval; SMD, standardized mean difference.

### Perioperative pain scores

In the unadjusted analysis, FLACC scores at rest and during activity at 2, 6, 12, and 24 h after surgery were all significantly lower in the GA + QLB group than in the GA group (all *P* < 0.001) (Table 4). After ICU postoperative analgesic strategy was incorporated into the mixed-effects models, the group-by-time interaction remained significant for both resting and active FLACC scores (both *P* < 0.001), indicating stable between-group differences in postoperative pain trajectories. In addition, an active analgesic strategy was independently associated with lower FLACC scores (rest: beta = −0.72, *P* = 0.012; activity: beta = −0.82, *P* = 0.007). Within 24 h after surgery, the proportions of infants receiving an active analgesic strategy were 20.5% (8/39) in the GA + QLB group and 10.8% (4/37) in the GA group, with no statistically significant difference (*P* = 0.348). Stratified sensitivity analyses showed that, in the conservative analgesia subgroup, the direction of the FLACC score differences at each time point was consistent with the main analysis; in the active analgesia subgroup, the direction was generally similar, although the statistical precision at 24 h was limited because the sample size was only 12.

**Table 4:** Postoperative analgesic strategy and FLACC pain scores.

Perioperative analgesic strategy			
Variable	GA + QLB group (*n* = 39)	GA group (*n* = 37)	*P* value
Active analgesic strategy within 24 h after surgery, n (%)	8 (20.5)	4 (10.8)	0.348
Panel A. FLACC scores at rest			
Time point	GA + QLB group (*n* = 39)	GA group (*n* = 37)	*P* value
2 h after surgery	2.00 (2.00, 2.00)	6.00 (5.00, 6.00)	< 0.001
6 h after surgery	2.00 (1.00, 2.00)	5.00 (4.00, 5.00)	< 0.001
12 h after surgery	1.00 (1.00, 1.00)	4.00 (3.00, 4.00)	< 0.001
24 h after surgery	1.00 (0.00, 1.00)	3.00 (2.00, 3.00)	< 0.001
Panel B. FLACC scores during activity			
Time point	GA + QLB group (*n* = 39)	GA group (*n* = 37)	*P* value
2 h after surgery	3.00 (3.00, 3.00)	7.00 (6.00, 7.00)	< 0.001
6 h after surgery	2.00 (2.00, 2.00)	6.00 (5.00, 6.00)	< 0.001
12 h after surgery	1.00 (1.00, 2.00)	5.00 (4.00, 5.00)	< 0.001
24 h after surgery	1.00 (1.00, 1.00)	4.00 (3.00, 4.00)	< 0.001

FLACC, face, legs, activity, cry, and consolability. Active analgesic strategy refers to postoperative opioid administration or continuous analgesic pump treatment in the ICU within 24 h after surgery.

### Perioperative complications and recovery

The incidence of PONV was significantly lower in the GA + QLB group than in the GA group [23.1% vs. 54.1%, *P* = 0.009]. After IPTW, GA + QLB was associated with a lower risk of PONV (RR, 0.40; 95% CI, 0.21–0.78); the multivariable model yielded a similar result (RR, 0.38; 95% CI, 0.21–0.72) (Table 5). There were no statistically significant differences between the groups in pruritus, postoperative complications, duration of urinary catheterization, time to recovery of enteral feeding, time to jaundice regression, length of hospital stay, or perioperative hypothermia (Table 6). No block-related complications, including local anesthetic systemic toxicity, puncture-site hematoma, or infection, were observed in the GA + QLB group.

**Table 5:** Incidence of postoperative nausea and vomiting (PONV).

Panel A. Unadjusted comparison			
Variable	GA + QLB group (*n* = 39)	GA group (*n* = 37)	*P* value
PONV, n (%)	9 (23.1)	20 (54.1)	0.009
Panel B. Adjusted analyses			
Model	Effect estimate	95% CI/balance	*P* value
Propensity score balance	All absolute SMDs after weighting < 0.10 (maximum 0.051)		
IPTW model	Adjusted RR 0.40	95% CI 0.21–0.78	0.007
Multivariable model	Adjusted RR 0.38	95% CI 0.21–0.72	0.003

PONV, postoperative nausea and vomiting; IPTW, inverse probability of treatment weighting; RR, risk ratio; CI, confidence interval; SMD, standardized mean difference.

**Table 6:** Postoperative recovery and complications.

Panel A. Recovery outcomes			
Variable	GA + QLB group (*n* = 39)	GA group (*n* = 37)	*P* value
Duration of urinary catheterization (d)	2.00 (2.00, 2.00)	2.00 (2.00, 2.00)	0.701
Time to recovery of enteral feeding (d)	3.00 (2.00, 3.00)	3.00 (2.00, 3.00)	0.565
Time to jaundice regression (d)	3.00 (3.00, 4.00)	3.00 (2.00, 6.00)	0.692
Length of hospital stay (d)	13.00 (12.00, 13.00)	13.00 (12.00, 13.00)	0.356
Panel B. Complications and adverse events			
Variable	GA + QLB group (*n* = 39)	GA group (*n* = 37)	*P* value
Perioperative hypothermia, n (%)	24 (61.5)	27 (73.0)	0.335
Pruritus, n (%)	5 (12.8)	11 (29.7)	0.094
Postoperative complications, n (%)	5 (12.8)	3 (8.1)	0.712

Data are presented as median (Q1, Q3) or n (%).

## Discussion

Using a single-center retrospective cohort design, this study evaluated, in a relatively large infant cohort, the potential value of ultrasound-guided bilateral quadratus lumborum block combined with general anesthesia during Kasai surgery. Compared with general anesthesia alone, this strategy was associated with faster postoperative extubation, lower intraoperative opioid exposure, lower FLACC scores within 24 h after surgery, and a lower incidence of postoperative nausea and vomiting. Importantly, these associations remained after inclusion of the ICU postoperative analgesic strategy in the mixed-effects model, as well as after IPTW and multivariable sensitivity analyses.

Previous studies have confirmed the advantage of epidural analgesia in reducing opioid use after Kasai surgery (9). Continuous epidural block can improve postoperative analgesia and reduce opioid use, and has advantages in early extubation and opioid sparing. However, in younger infants with biliary atresia, continuous epidural block still faces several issues: technical difficulty of neuraxial puncture, anesthesiologists’ concern regarding possible complications related to neuraxial puncture, difficulty in catheter care because infants cannot communicate verbally, and difficulty in confirming an effective sensory block level (10). All of these factors limit the widespread application of continuous epidural block. For abdominal wall plane blocks, external oblique intercostal block has been reported in case reports and small-sample experience after Kasai surgery, suggesting that it may be used for analgesia of upper abdominal incisions (11). However, the current amount of evidence remains limited, and whether it can provide relatively stable deep abdominal wall/visceral composite analgesia similar to that of QLB still requires future head-to-head comparison. In contrast, in young infants the abdominal wall musculature is thin, and the quadratus lumborum and the target fascial plane can be clearly displayed with a high-frequency linear probe. Bilateral QLB can be performed in the supine position, without moving the infant and changing body position as required for epidural puncture, which adds an extra risk of accidental tracheal tube dislodgement in small infants. In small infants undergoing anterior QLB, the needle path is short and there are no large blood vessels or vital organs along the path, so the technical difficulty of puncture is low and the risks of bleeding and hematoma caused by puncture are also relatively low.

In this study, the QLB group showed an approximately 22-min reduction in mean extubation time, significantly better pain scores at rest and during activity at multiple postoperative time points, and a markedly reduced incidence of nausea and vomiting. These findings are generally consistent with recently published pediatric QLB-related literature: QLB can reduce postoperative extubation time after pediatric abdominal surgery, lower rescue analgesic requirements, and improve early postoperative pain scores (6, 7, 12, 13). Randomized controlled trials have also indicated that the analgesic effect of QLB in lower abdominal surgery is superior to that of transversus abdominis plane block or caudal block (8, 14), and its advantage lies in the broader dermatomal coverage achieved by blocking the subcostal, iliohypogastric, and ilioinguinal nerves within the thoracolumbar fascial plane (4, 15). It is noteworthy that different approaches may affect block efficacy. Evidence suggests that anterior QLB is superior to lateral and posterior approaches in reducing postoperative fentanyl consumption and improving early pain control, implying that the diffusion pattern of the drug within the fascial plane may be a key variable determining the duration of analgesia (16). The potent analgesic effect observed in this study is highly consistent with the above literature, indicating that QLB can provide effective analgesia for Kasai surgery, significantly reduce dependence on opioids, and serve as an epidural analgesia alternative with a favorable safety profile.

From the findings of this study, it can be seen that because small infants, especially neonates, have incompletely developed liver and kidney function, continuous postoperative use of opioids carries a risk of respiratory depression (17, 18). ICU physicians therefore take a very cautious attitude toward postoperative opioid analgesia: among the 76 infants, only 12 (15.8%) received an active analgesic strategy, and among neonates the proportion was even lower, at 5.1% (2/39) receiving opioids for postoperative analgesia, which resulted in relatively high postoperative pain scores in this subgroup. Some studies have suggested that continuous epidural block for postoperative analgesia in children is relatively safe (10), and other studies have suggested that the long-acting local anesthetic liposomal bupivacaine may reduce postoperative pain in pediatric patients (19, 20). Considering the difficulty and procedural risk of epidural catheter placement in neonates, as well as the 20% risk of analgesic failure caused by postoperative catheter dislodgement (10), the use of liposomal bupivacaine for quadratus lumborum block in neonates may be considered in order to provide a longer duration of postoperative analgesia.

In this study, no significant between-group differences were observed in recovery-related secondary outcomes, including duration of urinary catheterization, time to recovery of enteral feeding, time to jaundice regression, length of hospital stay, perioperative hypothermia, pruritus, and overall postoperative complications. These findings may reflect the multifactorial nature of postoperative recovery in infants with biliary atresia, which is influenced not only by early analgesic quality and extubation status but also by disease severity, surgical trauma, feeding tolerance, and perioperative care pathways. Therefore, early analgesic advantages may not necessarily translate into measurable differences in all downstream recovery indicators in a retrospective cohort of this size.

This study used a retrospective cohort design and identified clinically meaningful associations, but causal inference remains limited. Baseline comparability was carefully assessed, and IPTW together with multivariable adjustment was used to reduce measured confounding; however, residual confounding and selection bias cannot be completely excluded. For example, QLB success could only be operationally defined according to ultrasound procedure records and the chart notation of “no block failure,” whereas sensory level and onset time were not uniformly available. In addition, as a single-center study, local anesthesia–surgery workflows, proficiency in ultrasound-guided block techniques, and postoperative care standards may limit generalizability. Although the sample size was sufficient to detect differences in the primary outcomes, it may have been underpowered for some safety indicators and secondary recovery outcomes, such as length of hospital stay.

In summary, this study suggests that, in infant Kasai surgery, quadratus lumborum block combined with general anesthesia is associated with shorter postoperative extubation time, lower opioid exposure, lower pain scores, and a lower incidence of postoperative nausea and vomiting, without evidence of block-related adverse events in this cohort. These findings support the potential effectiveness and feasibility of a regional block-based enhanced recovery strategy in this vulnerable population. Future prospective randomized studies are warranted to clarify causal effects and long-term outcomes using standardized QLB dosing, precise block-success assessment, and uniform postoperative analgesic protocols.

## Data Availability

The original contributions presented in the study are included in the article/Supplementary Material, further inquiries can be directed to the corresponding authors.
